# Efficient heavy metal ion removal by fluorographene nanochannel templated molecular sieve: a molecular dynamics simulation study

**DOI:** 10.1038/s41598-024-56908-3

**Published:** 2024-03-15

**Authors:** Youguan Ou, Zonglin Gu, Yuqi Luo

**Affiliations:** 1 Department of Gastrointestinal and Hepatobiliary Surgery, Shenzhen Longhua District Central Hospital, No. 187, Guanlan Road, Longhua District, Shenzhen, 518110 Guangdong Province China; 2 College of Physical Science and Technology, Yangzhou University, Jiangsu, 225009 China

**Keywords:** Fluorographene nanochannels, Membrane, Heavy metal ion removal, High efficiency, Atomic and molecular physics, Environmental chemistry, Nanoscience and technology

## Abstract

Environmental water contamination, particularly by heavy metal ions, has emerged as a worldwide concern due to their non-biodegradable nature and propensity to accumulate in soil and living organisms, posing a significant risk to human health. Therefore, the effective removal of heavy metal ions from wastewater is of utmost importance for both public health and environmental sustainability. In this study, we propose and design a membrane consisting of fluorographene (F-GRA) nanochannels to investigate its heavy metal ion removal capacity through molecular dynamics simulation. Although many previous studies have revealed the good performance of lamellar graphene membranes for desalination, how the zero-charged graphene functionalized by fluorine atoms (fully covered by negative charges) affects the heavy metal ion removal capacity is still unknown. Our F-GRA membrane exhibits an exceptional water permeability accompanied by an ideal heavy metal ion rejection rate. The superior performance of F-GRA membrane in removing heavy metal ions can be attributed to the negative charge of the F-GRA surface, which results in electrostatic attraction to positively charged ions that facilitates the optimal ion capture. Our analysis of the potential of mean force further reveals that water molecule exhibits the lowest free energy barrier relative to ions when passing through the F-GRA channel, indicating that water transport is energetically more favorable than ion. Additional simulations of lamellar graphene membranes show that graphene membranes have higher water permeabilities compared with F-GRA membranes, while robustly compromising the heavy meal ion rejection rates, and thus F-GRA membranes show better performances. Overall, our theoretical research offers a potential design approach of F-GRA membrane for heavy metal ions removal in future industrial wastewater treatment.

## Introduction

Water is a vital resource for the survival of humans, plants, and aquatic organisms. However, with the rapid development of industries, various types of wastewater, including organic and inorganic sewage, heavy metal ions, and radioactive wastewater, are generated^1–3^. Heavy metal ions, particularly presented in industrial wastewater, are highly toxic^4^, possessing a significant threat to the existing freshwater system when directly discharged into environment. This can directly or indirectly affect the health and safety of humans, plants, other organisms and even human health^5^. Heavy metal ions such as Pb^2+^, Hg^2+^, and Cd^2+^ are particularly toxic, and even when their concentrations do not exceed the specified limits, they pose a significant threat to the health of animals and plants. For example, Cd^2+^ exposure can cause bone rupture and kidney function damage^6–8^, and Pb^2+^ exposure can directly harm human brain cells, impair bone hematopoietic function, and induce anemia^9,10^. Similarly, Hg^2+^ exposure can cause severe kidney and bone damage, leading to bronchitis^11^. In particular, heavy metal accumulates in the body through the digestive system which presents serious harmful effect on the composition of gut microorganisms^12^. Due to the serious health consequences associated with exposure to these heavy metal ions, countries worldwide are taking significant measures to remove Pb^2+^, Hg^2+^, and Cd^2+^ from wastewater.

Various technologies and measures have been proposed to remove heavy metal ions from industrial wastewater, but these techniques have inherent limitations. Traditional heavy metal removal methods primarily rely on chemical precipitation and adsorption^13–16^. The chemical precipitation method employs counterions to limit the solubility of heavy metal ions in water and convert them into solid particles^17^, however, the treatment process generates toxic hydrogen sulfide vapor and wastewater containing residual sulfide^18^. The adsorption of metal ions on solid surfaces such as activated carbon is considered a competitive approach for separating metal ions from wastewater^19–21^. However, these methods are relatively high-cost and energy-intensive^22^.

In recent years, the use of nanomaterials has revolutionized the field of heavy metal wastewater treatment, particularly in the development of reverse osmosis membrane technology. This approach involves the passage of pure water through a reverse osmosis membrane that blocks the penetration of heavy metal ions simultaneously. Among the various types of nanomaterials used for this purpose, nanopores on the surface of nanomaterials, nanogaps formed at the edge of nanomaterials, and nanochannels formed on the surface of nanomaterials are the most widely studied^23–25^. Among these, nanopores and nanochannels have been found to be particularly effective^26^. For instance, Li et al. have demonstrated that functionalized graphene nanopores have a high heavy metal ion retention rate and fast water flux in treating heavy metal wastewater^23^. However, the precise control of the nanopores’ sizes in a large membrane aiming at achieving the best performance remains a challenge, and the permeability of water may be reduced compared to nanochannels. For example, the graphene nanopore can enlarge over time during the synthesis^27^, undoubtedly resulting in the altered pore, which thereby affects the performance of the membrane.

Fluorographene (F-GRA), a derivative of a layer of carbon sandwiched by two layers of fluorine atoms, exhibits exceptional physical and chemical properties, including a wide bandgap of 3.1 eV, the highest theoretical specific capacity of 865 mA h g^-1^, excellent thermal stability up to 400 °C, pronounced nonlinear characteristics, and high hydrophobicity^28,29^. Currently, F-GRA presents numerous applications in diverse fields, including lithium-ion batteries, nanoelectronics devices, light-emitting diodes, protective coatings, solid lubricants, nanocarriers, anticancer, and antibacterial agents^30–35^. Notably, the preparation methods of F-GRA, such as hydrothermal fluorination^36^ and gas fluorination^37,38^, have shown remarkable success. Despite these achievements, limited information is available on the performance of F-GRA in heavy metal ion removal. In addition, some previous studies^39,40^ have investigated the desalination capacity of lamellar graphene membranes, and found that the graphene membranes have been demonstrated possessing excellent performances. It should be noted that graphene is constructed by zero-charged carbon atoms. In comparison, F-GRA is fully covered by fluorine atoms at both basal surfaces, resulting in negatively charged surfaces of F-GRA. As yet, how the membranes consisting of such negatively charged surfaces affect the heavy metal ion removal remains unclear. To address this knowledge gap, we employed molecular dynamics simulation (MD) to establish F-GRA nanochannel based membrane by aligned several F-GRA sheets parallelly and investigated its efficacy in treating heavy metal wastewater. The simulations of lamellar graphene membranes reveal that graphene membranes have higher water permeabilities compared with F-GRA membranes, while robustly compromising the heavy meal ion rejections, and thus F-GRA membranes show better performances. Our simulation results demonstrate that F-GRA exhibits high water permeability and excellent rejection rate of heavy metal ions, thereby showing remarkable potential for industrial wastewater treatment.

## Method

### Molecular dynamics simulation

We utilized F-GRA to construct layered nanochannel filtration membranes. Specifically, we create an F-GRA sheet, which measured 5.95 × 6.91 nm^2^ in size. To create filter membranes, we stacked four layers of F-GRA nanosheets, with the layer spacing adjusted from 9 to 14 Å at 1 Å interval, based on the vertical distance of the nearest two fluorine atoms on two neighboring F-GRA nanosheets. These reverse osmosis membranes, composed of nanochannels, enable rapid water transport while impeding the passage of heavy metal ions (Cd^2+^, Hg^2+^, Pb^2+^). The other details of simulation boxes were showed in Table S1.

The Gromacs (version 2018) software package^41^ was used to simulate all systems in this study, and the VMD software^42,43^ was utilized to visualize and analyze all trajectories. The Charmm36 force field^44^ was employed, with the force field parameters of graphene and F-GRA obtained from previous studies^45,46^. The force field of F-GRA is also detailed in Table S2. Throughout the simulations, the F-GRA membrane was kept fixed, and to enhance sampling, three independent simulations were run in parallel for each system. The trajectory duration for each simulation was enough to calculate the salt rejection. Temperature was controlled at 300 K using a V-Rescale thermostat^47^, with periodic boundary conditions applied in all directions. Long-range electrostatic interactions were calculated using the PME method^48^, and the van der Waals interaction (vdW) was computed at a cutoff distance of 1.2 nm. The SETTLE algorithm was used to constrain the geometry of the water body^49^, with a time step of 2 fs employed during the simulation.

### Potential of mean force (PMF)

According to the umbrella sampling method^50–52^, the PMF value of water/Pb^2+^/Hg^2+^/Cd^2+^ from the wastewater to the F-GRA nanochannel was calculated. In detail, a water molecule, a Pb^2+^ ion, a Hg^2+^ ion, and a Cd^2+^ ion was pulled from the wastewater to F-GRA nanochannel interior along the direction perpendicular to the membrane surface via the formula: $w\left(r\right)=-{k}_{B}T{\text{ln}}g\left(r\right)$, wherein $w\left(r\right)$ indicated the PMF, ${k}_{B}$ and $T$ denoted boltzmann constant and temperature, $g\left(r\right)$ was the radial distribution function. During pulling process, the vertical distances (d) between the target molecule/ion to the membrane surface were kept at the references d_0_ via a harmonic force, F = K × (d–d_0_), where K was the force constant (3000 kJ mol^-1^ nm^-2^) and the sampling window was 0.1 nm. Such force constant was usually utilized previously^53–55^. At each d_0_, the system was balanced for 2 ns, followed by a 10 ns production run. The free energy profile was obtained by g_wham tools that implement the weighted Histogram analysis method^56,57^.

## Results

**Figure 1:**
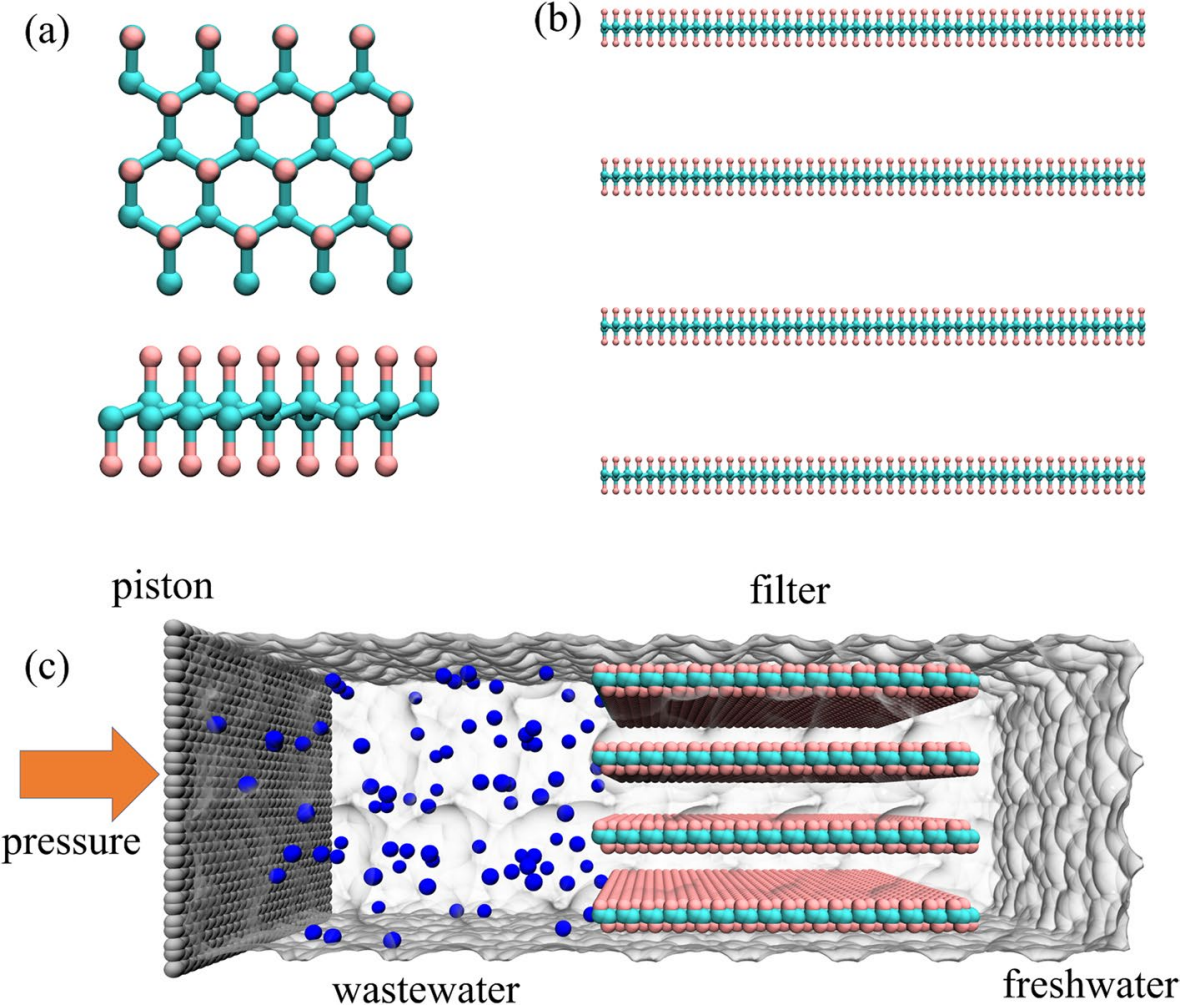
Initial configuration for purifying heavy metal wastewater using fluorographene (F-GRA) nanochannels. (**a**) Local top and side views of F-GRA. Cyan and pink spheres represent fluorine and carbon atoms, respectively. (**b**) Representative filtration membranes formed by four F-GRA nanosheets. (**c**) The configuration diagram of the heavy metal wastewater purification system, consisting of pistons (graphene nanosheets), heavy metal wastewater (including 0.5 M CdCl_2_, HgCl_2_, or PbCl_2_), F-GRA filter membrane, and fresh water. The water boundaries are depicted as transparent surfaces.

Figure 1a depicts a local top and side view of F-GRA, where fluorine atoms are evenly distributed on both basal planes of the carbon layer, and all carbon atoms exhibit sp^3^ hybridization. By stacking four F-GRA nanosheets, we create nanochannels based membrane with varied interlayer spacings, as shown in Fig. 1b. The interlayer spacing between adjacent F-GRA nanosheets was adjusted from 9 to 14 Å to investigate the effect of interlayer spacing on the purification performance of heavy metal ion wastewater. The simulated system, as representatively illustrated in Fig. 1c, comprises four key modules: a piston composed of graphene nanosheet, heavy metal wastewater consisting of 0.5 M CdCl_2_, HgCl_2_ or PbCl_2_, filter membranes (F-GRA nanochannels), and fresh water.

**Figure 2:**
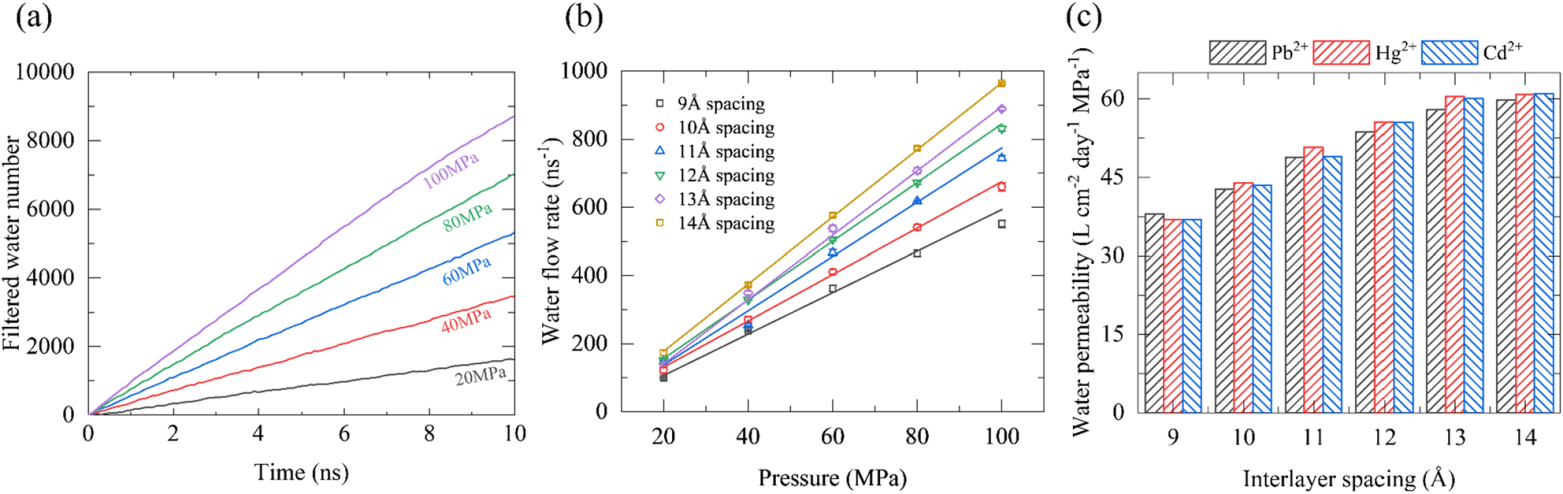
Water permeability through F-GRA membrane. (**a**) Time-dependent filtered water number in the HgCl_2_ system under the 13 Å interlayer spacing. (**b**) The water flow rate with different layer spacings at different pressures. (**c**) Average water permeability through F-GRA channels with different layer spacings.

Figure 2a displays the accumulated permeated water molecules through the channel as a function of simulated time at five different pressures (using Hg^2+^ as an example). In this study, the quantity of permeated water is calculated as the number of filtered water, which is the difference between the number of water on the freshwater region at t ns and 0 ns (i.e., N_t_ − N_0_). Clearly, the amount of permeated water number increases linearly with the simulation time extension. The curve becomes steeper with the rise of external pressure, that is, higher pressure enables faster water permeation. We computed the corresponding water flow rate (as shown in Fig. 2b) based on the slopes of the water filtered number curves. The water flow rate through F-GRA membrane is closely related to the applied external pressure and the layer spacing of the nanochannels. At the same interlayer spacing, the rate of water flow through the channel also shows a linear relationship with the applied pressure. At the same pressure, the greater the layer spacing of the channel, the faster the water permeation. Other systems of Pb^2+^ and Cd^2+^ as shown in Fig. S1 also present the same trend. This is a result of that enlarged interlayer spacing has larger interspace that allows more water to pass across. The water permeability of the F-GRA membrane is a crucial parameter for evaluating the effectiveness of the membrane. The results (Fig. 2c) indicate that the water permeability increases gradually as the layer spacing of the F-GRA nanochannels increases from 9 to 14 Å, regardless of rejected ion species. In particular, at an interlayer spacing of 14 Å, the water permeability of Cd^2+^, Hg^2+^, and Pb^2+^ systems reach up to 60.09, 60.92, and 59.82 L cm^-2^ day^-1^ MPa^-1^, respectively. This trend is in line with the expectation that a larger channel volume provides more space to accommodate more water molecules, leading to faster water flow. Overall, these findings demonstrate that F-GRA membrane has excellent water permeability in heavy metal wastewater purification.

**Figure 3:**
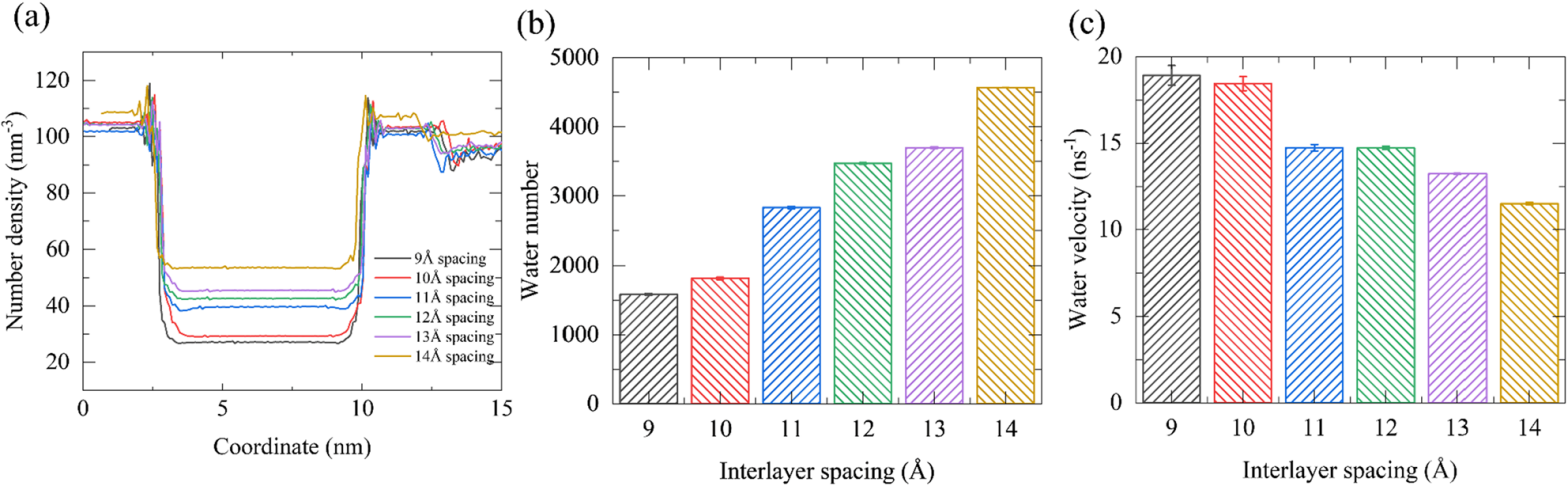
Characteristics of water within F-GRA nanochannels. (**a**) Local axial distribution of water density inside F-GRA nanochannels. (**b**) Average number of waters in the F-GRA nanochannel interior. (**c**) Averaged axial velocity of water molecules within nanochannels. All data were collected from three parallel simulations of 100 MPa.

The characteristics of water molecules within the channel are closely associated with the filter membrane permeability. In order to explore this relationship, we analyzed the axial distribution of water density in the F-GRA channel using the Hg^2+^ system as an example (Fig. 3a). The water density inside the membrane channels increases as the interlayer spacing varying from 9 to 14 Å, and the same pattern is observed in the Cd^2+^ and Pb^2+^ systems (Fig. S2). This is the main reason for the difference in permeability among the six different membranes. The transverse distribution of water density confined in the F-GRA channels was also examined (Fig. S3). In the 9 Å channel, the water molecules show the highest density as a single strand, resembling a single leaf as they pass through the F-GRA channel. However, in the 10 Å to 12 Å channels, the single strand gradually splits into two chains that are separately close to the two adjacent F-GRA surfaces, with a tendency to form three chains at 13 Å and three layers of water chains formally generated in the 14 Å channel. Moreover, the average number of water molecules inside channels with different layer spacings indicates that the larger the layer spacing, the more water molecules can be accommodated (Fig. 3b). However, the water velocity trend is opposite to the layer spacing, with a decrease in velocity following the layer spacing increase (Fig. 3c). This is resulted from that 9 Å spacing can accommodate a single layer of waters to move, whereas in the 10–14 Å, there are more water layers (two or three layers) of water leaves (Fig. S4), leading to enhanced the internal interaction within water layers that reduces the water flow velocity. However, the decrease in water velocity from 9 to 14 Å does not weaken the water flow. On the contrary, due to the improved volume of channel interior from 10 to 14 Å, which can accommodate two to three layers of water leaves, the water flow gradually strengthens. Overall, these results also confirm the permeability as shown in Fig. 2.

**Figure 4:**
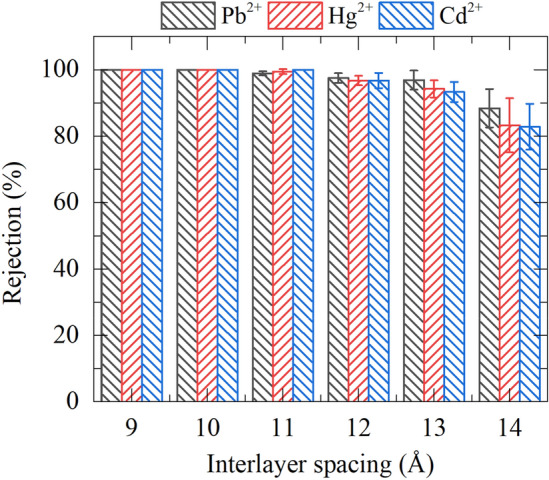
Heavy metal ion rejection rates of F-GRA nanochannels at different layer spacings (in CdCl_2_, HgCl_2_, and PbCl_2_ systems).

**Figure 5:**
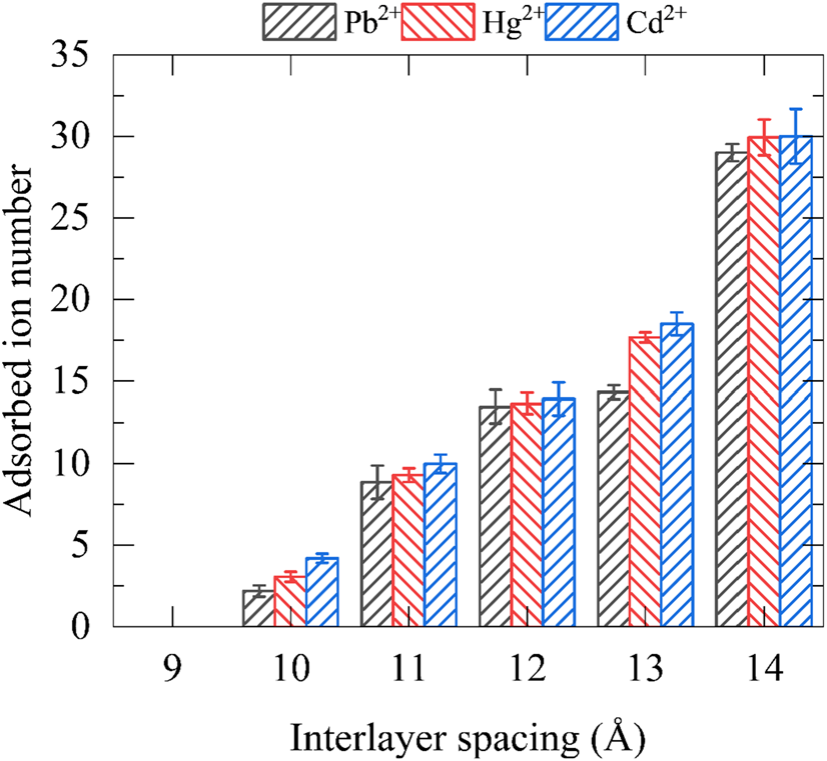
Number of ions adsorbed in the nanochannel interior at different interlayer spacings.

In addition to water permeability, the rejection rate of heavy metal ions is another crucial indicator of the purification effectiveness of heavy metal ion wastewater (Fig. 4). To calculate the ion rejection rate, we adopt the formula^58,59^: heavy metal ion rejection = $\left(1-\frac{{C}_{p}}{{C}_{f}}\right)\times 100\%$, where ${C}_{p}$ indicates the concentration of heavy metal ions in permeation chamber when half of the water on the wastewater region passes through the filter membrane, and ${C}_{f}$ denotes the concentration of heavy metal ions in feed chamber at 0 ns. Noticeably, the interception rates of heavy metal ions in most systems exhibit desirable values with most over 90%, and even close to 100% in cases of 9 ~ 11 Å interlayer spacing. Although the rejection is slightly declined after the spacing prolonged to 14 Å, the corresponding values are still ideal (> 80%). These results indicate that the F-GRA membrane utilized herein can effectively impede the passage of heavy metal ions, realizing the purification goal of heavy metal ion wastewater. The Movie S1 also depicts the effective separation of heavy metal ions. We then calculated the heavy metal ions adsorbed inside the F-GRA channels as shown in Fig. 5. It is noteworthy that no ion can adsorb in 9 Å channels, implying that in 9 Å interlayer spacing, the heavy metal ion rejection of F-GRA membrane is attributed to the steric repulsion effect. In other words, hydrated heavy metal ion is incapable of entering into the 9 Å sized F-GRA channels. Nevertheless, from 10 to 14 Å, the adsorbed heavy metal ion number in the F-GRA interior becomes larger. That is to say, after the interlayer spacing extended, the steric repulsion effect is continuously weakened and the adsorption separation mechanism becomes more important. Further calculations (Figure S5) show that the electrostatic attraction between positively charged heavy metal ions and negatively charged F-GRA surface dominantly mediates the robust adsorption of ions inside the F-GRA channels. In addition, in our simulations, due to the short simulation time, we cannot observe the clear hindrance of adsorbed ions to water speed. However, in realistic long-term sieving process, such ion adsorption will undoubtedly influence the water transport.

**Figure 6:**
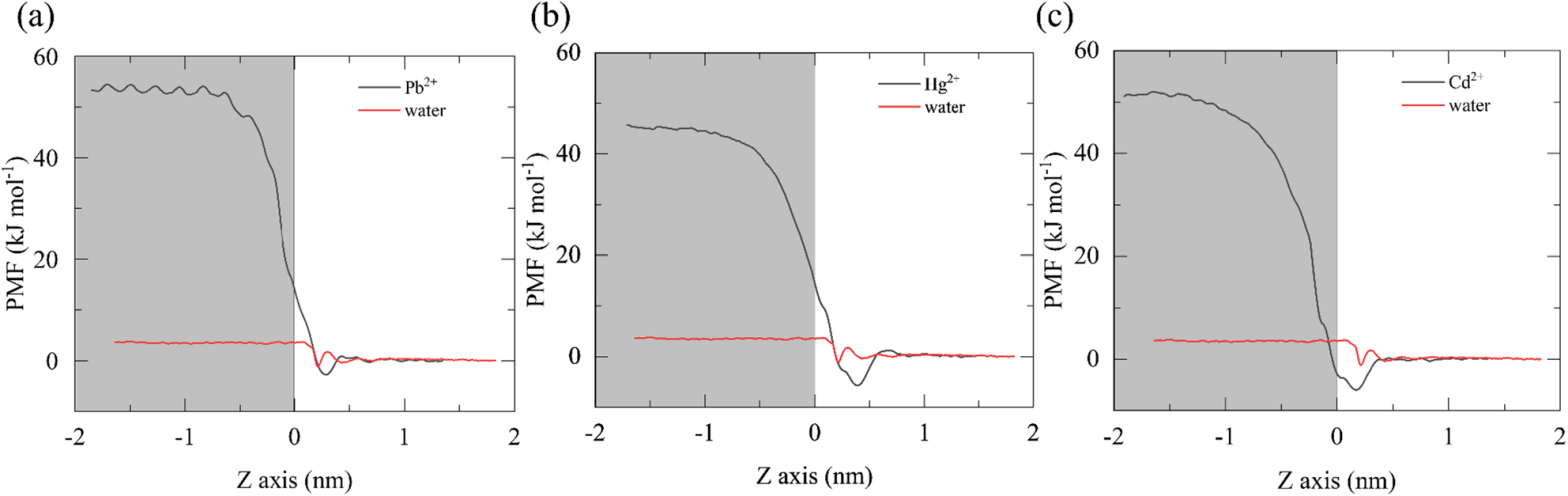
The potential of mean force by pulling a water molecule, a Pb^2+^, a Hg^2+^, a Cd^2+^ from the wastewater side to the interior of the 9 Å channel. Gray background indicates the interior of the channel, while white background indicates the wastewater region.

In order to deeply reveal the superior performance of F-GRA membrane in removing heavy metal ion, we performed free energy calculations based on the potential of mean force (PMF) by pulling a water molecule, Cd^2+^, Hg^2+^ and Pb^2+^ migrating from the heavy metal ion wastewater region to the membrane interior (Fig. 6). Obviously, when entering into the F-GRA nanochannel, water molecule experiences the smallest energy barrier whereas the energy barriers of three heavy metal ions completely exceed the one of water. This indicates that water molecules can pass through the F-GRA membrane more easily than heavy metal ions, allowing for rapid water flow while preventing the passage of heavy metal ions. In addition, the size exclusion effect^60^ of hydrated ions by the membrane is responsible for the high free energy of heavy metal ions. That is to say, heavy metal ions can form hydrated ions via their strong electrostatic attraction. When it pass through the membrane, heavy metal ion traverses either accompanied by its hydrated waters, or via stripping off some hydrated waters aiming to reducing its size to fit the nanochannel’s dimension. However, stripping off the hydrated waters around heavy metal ion should cost energy against the strong electrostatic attraction between ion and hydrated waters. Moreover, due to the robust adsorption of heavy metal ions inside the nanochannels (as discussed above and also see Fig. 5), the efficient heavy metal ion separation is attributed to the interplay of the size exclusion effect and ion adsorption. In detail, at small F-GRA nanochannels (9 Å), the heavy metal ion rejections are completely rejected due to the larger size of hydrated ion than F-GRA nanochannel. When the interlayer increases (> 9 Å), partial heavy metal ions enter into membrane through stripping off few hydrated waters, and then adsorb in the nanochannels. Therefore, the permeation of water through F-GRA membrane is energetically more favorable than heavy metal ions. Further PMF profile by pulling a typical Pb^2+^ through a 13 Å interlayer spacing (Figure S6) shows that the energy barrier of heavy metal ion is sharply reduced, which hints that Pb^2+^ is able to transfer through F-GRA channels more easily after augmenting the interlayer spacing. Hence, in large interlayer spaced F-GRA membrane (e.g., 14 Å), the adsorption mediated heavy metal ions removal is more significant to the high salt rejection capacity of F-GRA membrane (as discussed above).

We also compare the separation capacity of F-GRA nanochannel membrane with previously reported membranes as summarized in Table S3. The membranes based on MoS_2_ and boron nitride nanopores^61,62^ show the largest heavy metal ion removal rate of 100%, although their water permeabilities are ~ 10 and 15.15 L cm^-2^ day^-1^ MPa^-1^, respectively. The boron atom functionalized graphene nanopore^63^ has the lowest heavy metal ion removal rate, which possesses ~ 16.5 L cm^-2^ day^-1^ MPa^-1^ water permeability. In comparison, the F-GRA nanochannel based membrane features the best performance with its water permeability 3.6–6 times larger than other reported membranes, and with the heavy metal ion removal rate approaching over 90%. Therefore, the F-GRA membrane is a good candidate for heavy metal ion removal from wastewater.

Previously, many studies^39,40^ have explored the desalination capacity of lamellar graphene membranes, by which the graphene membranes have been demonstrated possessing excellent performances. However, the heavy metal ion removal capacity of lamellar graphene membranes is never investigated and thus the performance of F-GRA and graphene membranes can not be directly compared. Therefore, we conducted more simulations to evaluate the heavy metal ion removal capacity of lamellar graphene membranes as shown in Figure S7. We constructed the simulation system of graphene membranes similar to Fig. 1c, wherein the F-GRA membranes were replaced by the graphene membranes. Herein, we chose the Cd^2+^ heavy metal ion as an example. We note that the water permeabilities at each membrane show fluctuated value around ~ 60 L cm^-2^ day^-1^ MPa^-1^. The graphene membranes possess higher water permeability than F-GRA membranes at the interlayer spacings of 9–12 Å, although the water permeabilities of two membranes at 13–14 Å interlayer spacings are comparable. However, the heavy metal ion rejection rates of graphene membranes are 92.3%, 86.6%, 85.5%, 75.5%, 72.1% and 71.9%, respectively. In comparison with F-GRA membranes, the graphene membranes have much lower ion rejection rates, indicating their weaker heavy metal ion rejection capacity. Overall, considering the significance of heavy metal ion rejection, we believe that F-GRA membranes have a better performance than graphene membranes.

## Conclusion

In conclusion, we employed molecular dynamics simulations to investigate the efficacy of F-GRA nanochannel based membrane in removing heavy metal ions. Our findings demonstrate that the F-GRA channel exhibits favorable heavy ion retention and high water flux. The water flow rate is governed by the external pressure and interlayer spacing, whereby an increase in the layer-separation distance and pressure leads to enhanced water flow. Notably, the water permeability of F-GRA ranges between 36 and 60 L cm^-2^ day^-1^ MPa^-1^, which represents a significant advantage in wastewater treatment. The distribution of water within the F-GRA channel reveals that larger spacing facilitate greater water flow. Additionally, water molecules tend to form a chain within the 9 Å layer spacing, and a propensity towards dual water chains was observed at 10 Å, while water molecules produced dual chains within the 11 Å, 12 Å layers. At 13 Å, a tendency to form three water chains was observed, while three chains were formed at the 14 Å layer spacing. Moreover, the rejection rates of heavy metal ions achieved in all simulations are very promising and those in the 9–11 Å systems can even reach 100%. The adsorption separation mechanism is more distinct when the interlayer spacing is up-regulated, whereas the steric repulsion effect works in small interlayer spacing. Furthermore, the free energy profiles based on the average force potential corroborate that the free energy barrier follows the order of heavy metal ions > water, signifying that water molecules traverse the F-GRA channel with greater ease than heavy metal ions, thereby contributing to the high water permeability of F-GRA. We also performed more simulations of lamellar graphene membranes, wherein graphene membranes have higher water permeabilities compared with F-GRA membranes, while their corresponding heavy metal ion rejection rates are much lower. Given that the ion rejection is the most major goal of separation membrane, we believe that F-GRA membranes have a better capacity than graphene membranes. These findings highlight the efficient removal of heavy metal ions by F-GRA membrane, with implications of the potential usage in future.

### Supplementary Information

Supplementary Video 1.

Supplementary Information 1.

## Data Availability

The datasets used and/or analysed during the current study available from the corresponding author on reasonable request.
