# Supplementary material for: Efficient heavy metal ion removal by fluorographene nanochannel templated molecular sieve: a molecular dynamics simulation study
Source: Sci Rep. 2024 Mar 15;14:6298. doi: 10.1038/s41598-024-56908-3 (PMC10943243; doi:10.1038/s41598-024-56908-3)
Supplement: Supplementary file 2 — Supplementary Information 1. [file 41598_2024_56908_MOESM2_ESM.docx]

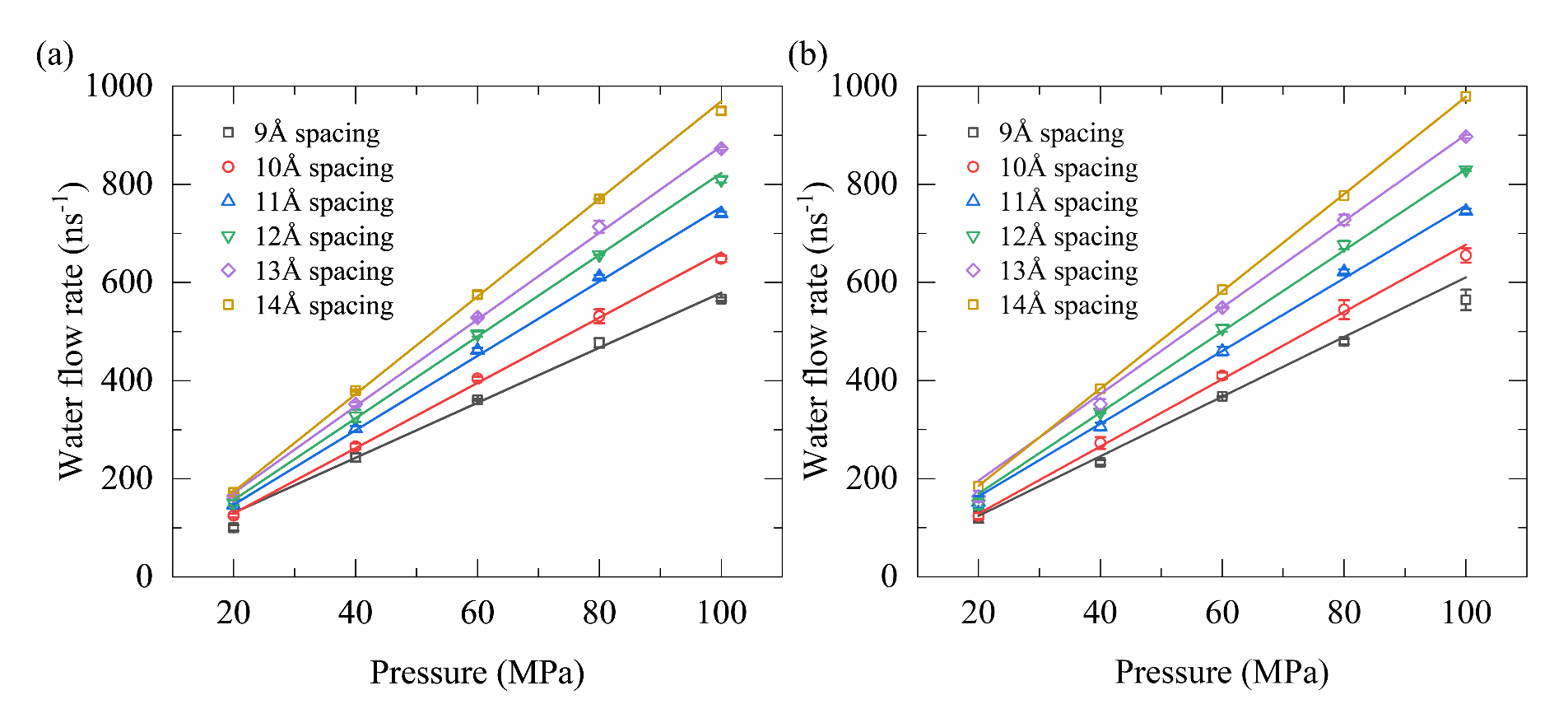


Figure S1. The flow rate of F-GRA channels with different layer spacings at different pressures. (a) the PbCl_2_ system and (b) the CdCl_2_ system.


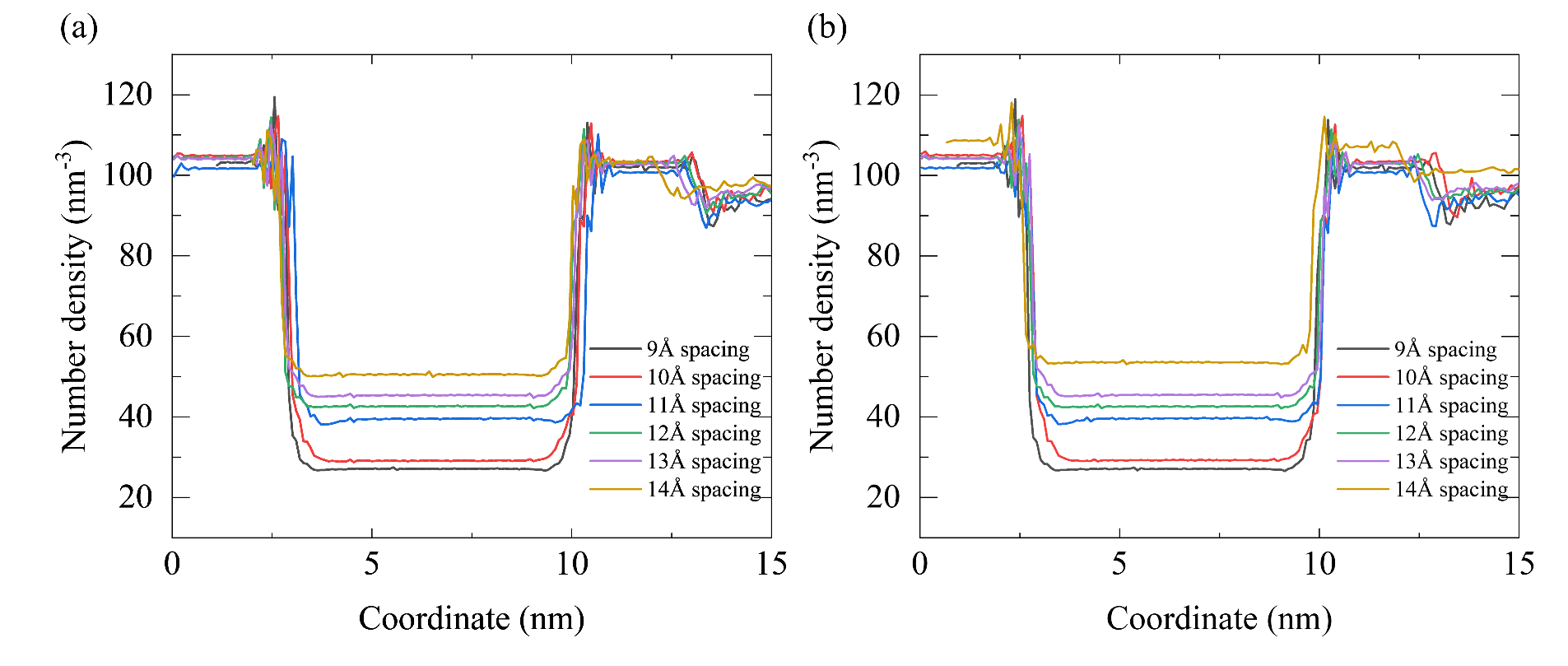


Figure S2. The local axial distribution of water density within the F-GRA nanochannel. (a) the PbCl_2_ system and (b) the CdCl_2_ system.


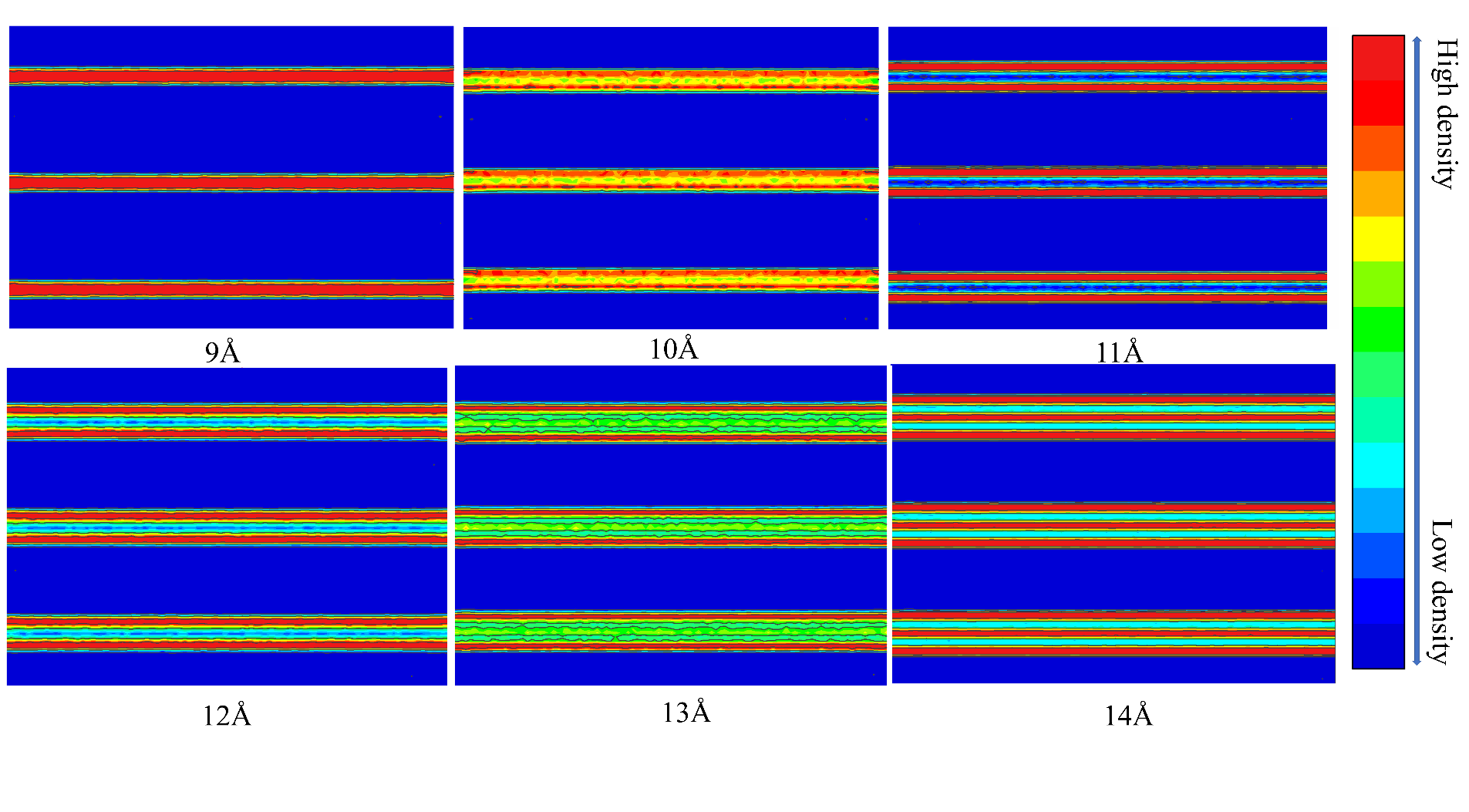


Figure S3. Transverse distribution of water densities inside F-GRA nanochannels with 6 different layer spacings at the same pressure of 100 MPa.


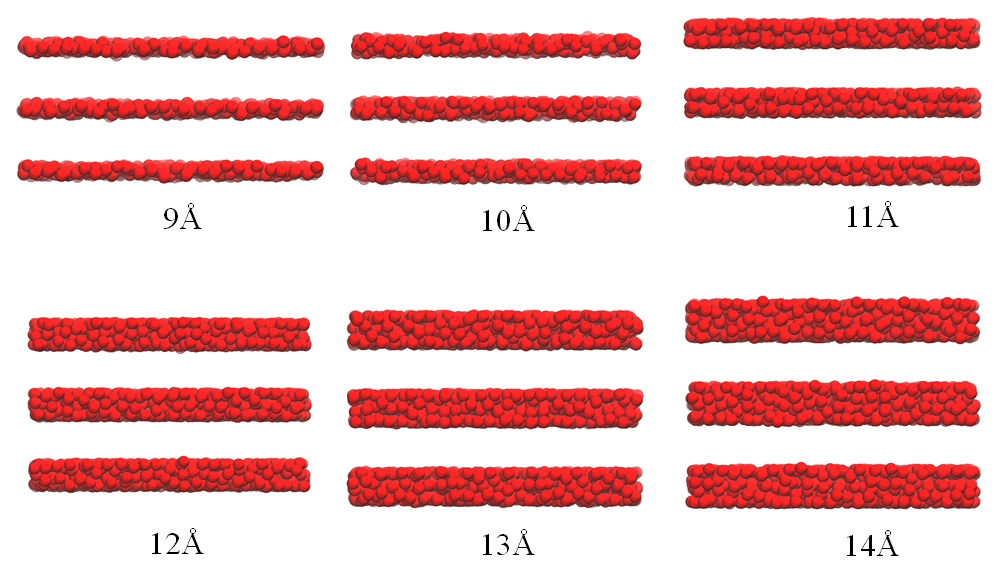


Figure S4. Configurations of oxygen atoms of water molecules within F-GRA nanochannels of 9 ~ 14 Å layer spacing.


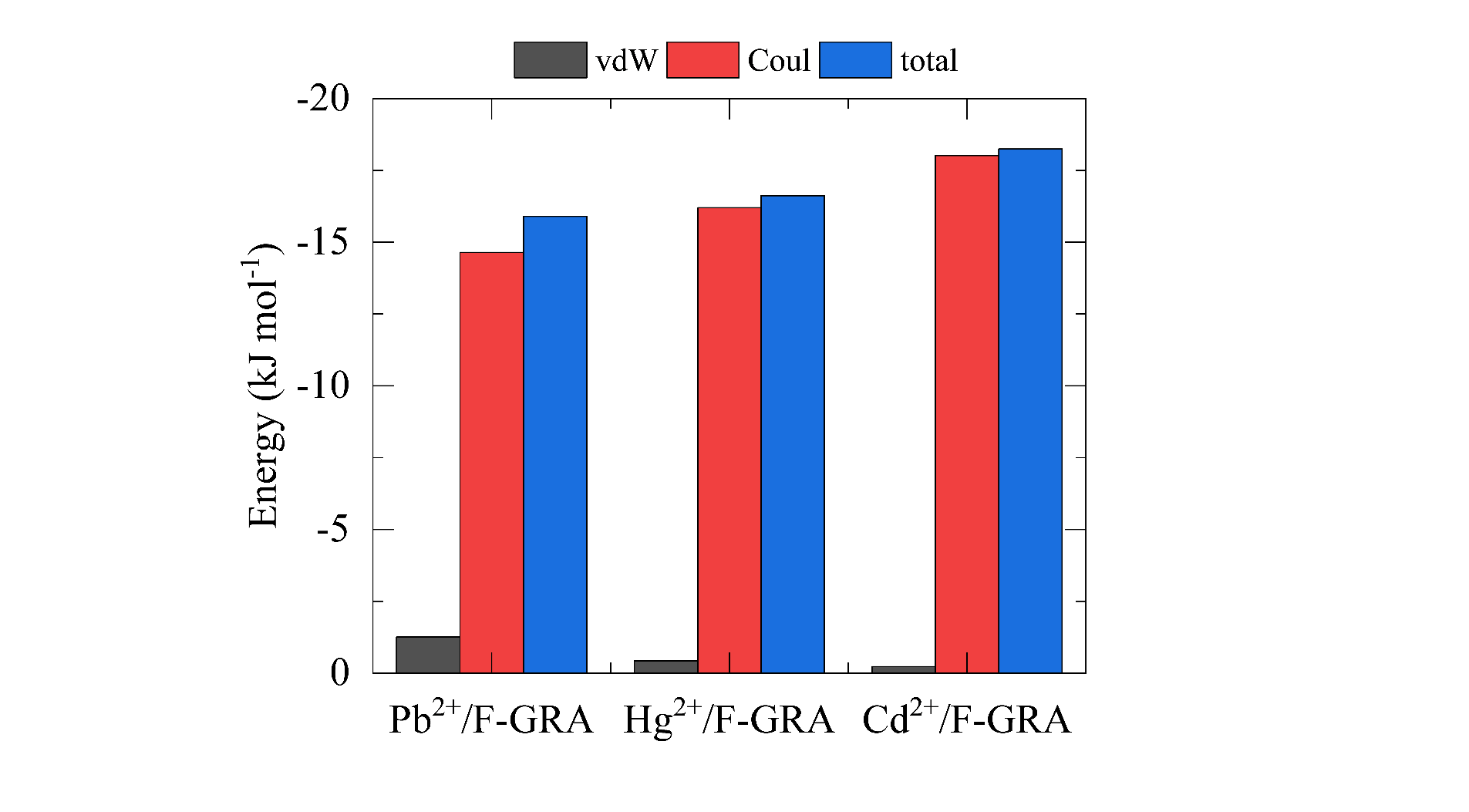


Figure S5. Averaged interaction energy (including vdW, coulomb, and total energy) between Pb^2+^, Hg^2+^, and Cd^2+^ and F-GRA. These data are obtained from Pb^2+^, Hg^2+^, and Cd^2+^ in the F-GRA channel interior with an interlayer spacing of 13 Å.


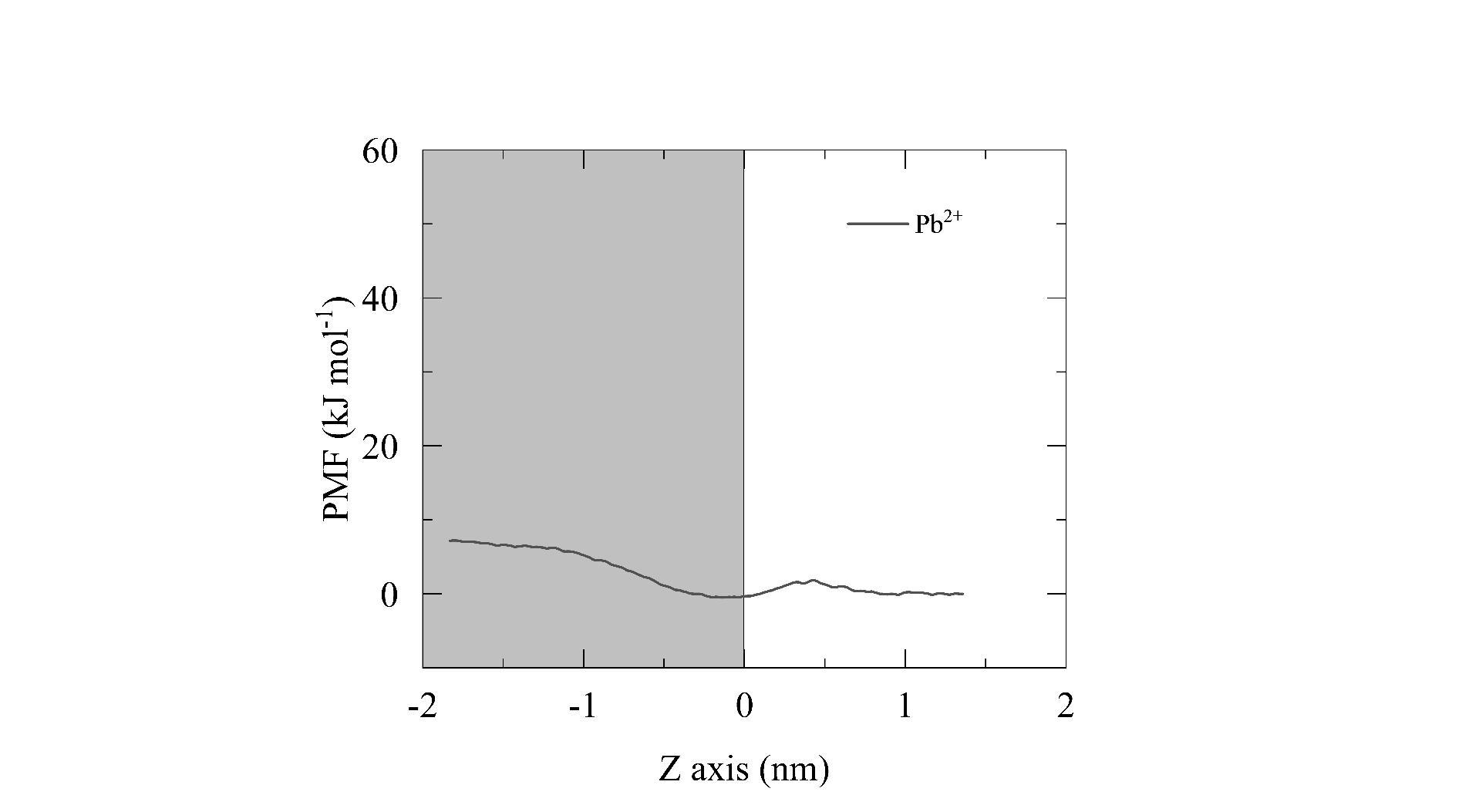


Figure S6: The potential of mean force by pulling Pb^2+^ from wastewater to the 13 Å channel interior.


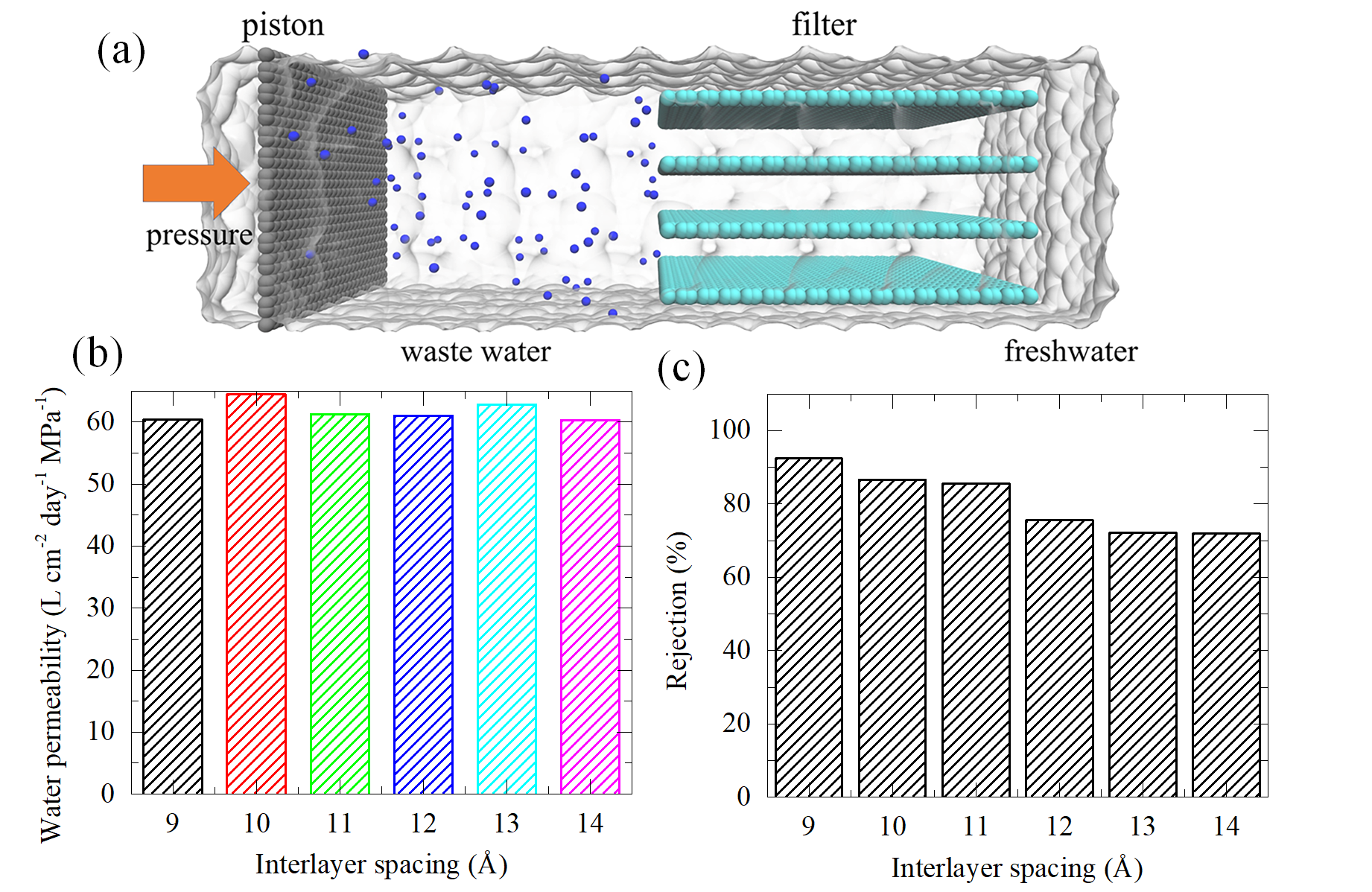


Figure S7. Separation capacity of lamellar graphene membrane for Hg^2+^ solution. (a) Simulation setup. The simulation setup is similar to Figure 1c, wherein the F-GRA filter membrane is replaced by lamellar graphene membrane. (b) Water permeability of graphene membrane with different interlayer spacings. (c) Heavy metal ion rejection rates of different membranes.

Movie S1. Representative movie of separation process of F-GRA membrane with 9 Å interlayer spacing under 250 MPa.

Table S1. Details of simulation boxes.

| Interlayer spacing (Å) | 9 | 10 | 11 | 12 | 13 |  | 14 |
| --- | --- | --- | --- | --- | --- | --- | --- |
| box size (nm^3^) | 6.0×5.0×18 | 6.0×5.4×18 | 6.0×5.8×18 | 6.0×6.2×18 | 6.0×6.6×18 |  | 6.0×7.0×18 |
| Water molecule number | 12682 | 14089 | 15553 | 17241 | 18610 |  | 20300 |
| Heavy metal ion number | 70 | 77 | 82 | 90 | 94 |  | 101 |

Table S2. Force fields between F-GRA and water. i and O indicate the fluorine/carbon atoms and oxygen atom of water molecule, respectively.

| atom name | $\sigma$i-O (Å) | $\varepsilon$i-O (kJ/mol) | charge (e) |  |
| --- | --- | --- | --- | --- |
| i = fluorine | 4.231 | 0.220 | -0.56 |  |
| i = carbon | 3.436 | 0.350 | 0.56 |  |

Table S3. Separation capacity comparison of F-GRA nanochannel membrane and previously reported membranes.

|  | water permeability (L cm^-2^ day^-1^ MPa^-1^) | heavy metal ion removal rate |
| --- | --- | --- |
| F-GRA nanochannel (13 Å interlayer spacing) | ~ 60 | over 90% |
| boron atom functionalized graphene nanopore[[1](#_ENREF_1)] | ~ 16.5 | over 80% |
| MoS_2_ nanopore[[2](#_ENREF_2)] | ~ 10 | 100% |
| boron nitride nanopore[[3](#_ENREF_3)] | 15.15 | 100% |

Reference

[1] Y.P. Li, Z.J. Xu, S.Y. Liu, J.W. Zhang, X.N. Yang, Molecular simulation of reverse osmosis for heavy metal ions using functionalized nanoporous graphenes, Comput. Mater. Sci. , 139 (2017) 65-74.

[2] J. Azamat, A. Khataee, Improving the performance of heavy metal separation from water using MoS2 membrane: Molecular dynamics simulation, Comput. Mater. Sci. , 137 (2017) 201-207.

[3] J.B. Du, Z.F. Feng, L.J. Han, X.Y. Ma, Q.F. Li, Understanding the water permeability and Cu2+ removal capability of two-dimensional nanoporous boron nitride, Comput. Mater. Sci. , 184 (2020) 109923.
